# Physiological effects of hand grip and cold pressor tests in young Saudi adults

**DOI:** 10.25122/jml-2024-0378

**Published:** 2024-12

**Authors:** Raju Suresh Kumar, Abdulaziz Abdullah Alqarni, Musaad Jamaan Alghamdi, Saad Amer Alharbi, Omar Loutfi Alsharef, Mohamed Eldigire Ahmed

**Affiliations:** 1 Department of Basic Sciences, College of Science and Health Professions (COSHP), King Saud bin Abdulaziz University for Health Sciences (KSAU-HS), Jeddah, Saudi Arabia; 2 King Abdullah International Medical Research Center (KAIMRC), Jeddah, Saudi Arabia; 3 Department of Respiratory Therapy, College of Applied Medical Sciences (COAMS), King Saud bin Abdulaziz University for Health Sciences (KSAU-HS), Jeddah, Saudi Arabia

**Keywords:** blood pressure, cold pressor test, heart rate, isometric hand grip, respiratory rate, BP, Blood Pressure, HR, Heart Rate, RR, Respiratory Rate, SpO2, Peripheral Arterial Oxygen Saturation, ANS, Autonomic Nervous System, IHT, Isometric Handgrip Test, CPT, Cold Pressor Test, SBP, Systolic Blood Pressure, DBP, Diastolic Blood Pressure, MAP, Mean Arterial Pressure, BMI, Body Mass Index

## Abstract

The risk of cardiovascular disease differs among various ethnic groups, highlighting disparities in cardiovascular health among different populations. While multiple studies from other countries have looked at changes in physiological parameters during autonomic function tests like isometric handgrip and cold pressor tests, no correlational research has been done in Saudi Arabia. This lacuna underscores the importance of examining the relationship between cardiorespiratory parameters in young Saudi Arabian individuals during these tests. This study aimed to determine the correlation between the isometric handgrip and cold pressor tests and physiological parameters in healthy young Saudi Arabian college students. A single-arm interventional study was conducted with a cohort of 65 healthy young adult Saudi college students, including male and female participants. A point estimate was calculated with a 95% confidence level. Physiological parameters were analyzed and compared at rest and during isometric handgrip and cold pressor tests. The study involved participants with an average age of 21.12 ± 1.02, predominantly male students. A significant impact was observed only in respiratory rate (*P* = 0.007) during the isometric handgrip and cold pressor tests. In contrast, blood pressure parameters and arterial oxygen saturation values showed no statistical significance during both tests. This sheds light on their autonomic responses to physiological stressors and contributes to our understanding of cardiovascular health across diverse populations, guiding future interventions for global improvements in cardiorespiratory outcomes.

## INTRODUCTION

Vital indicators like blood pressure (BP), heart rate (HR), respiratory rate (RR), and peripheral arterial oxygen saturation (SpO_2_) are fundamental markers of physiological functioning [1,2]. It is well-documented that the autonomic nervous system (ANS) plays a critical role in regulating cardiovascular functions [3]. ANS adjusts heart rate and regulates blood pressure to meet the demands of daily activities [4]. Hadaya *et al*. [5] reported that autonomic nervous system dysfunction is associated with the onset of cardiovascular diseases. Researchers have reported that ANS is involved in the modulation of RR [3]. Therefore, autonomic function tests are essential in evaluating and identifying changes in normal cardiorespiratory function.

The isometric handgrip test (IHT) is widely used to assess cardiovascular autonomic functions [6,7]. The cold pressor test (CPT), a well-established autonomic function test, is recognized for inducing significant cardiovascular responses by activating the sympathetic nervous system, resulting in elevated HR and BP. It has been employed as a research tool to assess the integrity of ANS [8]. A review by Chong *et al*. [9] indicated racial differences in sympathetic nervous system functioning. Furthermore, ethnic disparities and psychosocial factors are linked to variations in autonomic nervous system function [10]. Gender and racial differences in autonomic cardiovascular regulation have been reported, which may contribute to disparities in susceptibility to hypertension and cardiovascular diseases [11].

Physiological parameters like systolic blood pressure (SBP), diastolic blood pressure (DBP), mean arterial blood pressure (MAP), HR, RR, and SpO_2_ during CPT and IHT have not been previously studied in the Saudi Arabian population. This gap in the literature underscores the importance of understanding physiological variations across different ethnic groups. This could offer crucial insights for customizing interventions and shaping future cardiorespiratory research. Therefore, this study aimed to examine the cardiorespiratory responses to autonomic function tests, specifically IHT and CPT, by evaluating physiological parameters in healthy young Saudi Arabian adults.

## MATERIAL AND METHODS

### Study design, setting, and population

This single-center, single-arm interventional study was conducted among young, healthy male and female students from various health sciences disciplines at King Saud bin Abdulaziz University for Health Sciences, Jeddah, Saudi Arabia. A total of 253 students, aged 19 to 24, were screened. The inclusion criteria focused on selecting Saudi Arabian college students in good health with no history of prior illnesses. Exclusion criteria included individuals with hypertension, upper limb injuries or weakness, motor neuron diseases, diabetes mellitus, respiratory diseases, smokers, those who were fasting, and pregnant individuals. After applying the inclusion and exclusion criteria, a final sample of 65 participants (45 men and 20 women) was selected. The study occurred between October 2020 and October 2021 and received approval from the institutional bioethics committee and the review board. Participants were chosen using consecutive sampling, a non-probability technique for selecting individuals until the required sample size is achieved. The required sample size was estimated using the following formula:


$$n=\frac{Z^{2}\sigma^{2}}{e^{2}}$$

Where ***n*** is the sample size

***z*** is the critical value at a 95% confidence level

***e*** is the margin of error (2%). The standard deviation of HR was estimated at 8.14 for the normal population using previous similar studies. The required sample size was calculated using a margin of error (e) of 2, as follows:


$$1.96^{2}*\;8.14^{2}/2^{2}=\;65$$

The research complied with the ethical standards set by the institutional research committee following the 2013 Helsinki Declaration. All participants gave explicit, written consent before the study began, and their privacy was safeguarded with the utmost care throughout the study.

### Study procedure

Baseline demographic information was collected for each participant, including height, weight, and body mass index (BMI). The body mass index (BMI) was calculated using the World Health Organization (WHO) guidelines [12]. Physiological parameters, including SBP, DBP, MAP, HR, RR, and SpO_2_, were evaluated during rest periods and while performing two specific tests: the IHT and CPT. The IHT was performed using an isometric handgrip device (Generic). SBP, DBP, and HR were monitored using an automated sphygmomanometer (Omron M2 Intelli IT), and SpO_2_ was measured with a pulse oximeter (Tomorotec). Two trained researchers observed and manually tallied the respiratory rates to ensure consistency and minimize discrepancies by counting the instances of the subject's chest or abdomen rising within one minute [13].

### Isometric handgrip test (IHT)

The IHT was conducted using the subject's dominant hand. Simultaneously, a blood pressure cuff was securely placed on the upper arm of the non-dominant hand, positioned over the brachial artery. SBP, DBP, MAP, HR, RR, and SpO_2_ were continuously monitored from the non-dominant hand. Participants were instructed to maintain an isometric grip with their dominant hand, supported on a table, ensuring the wrist remained in a neutral position and the elbow flexed at a 90° angle [14]. They were asked to perform a single maximum voluntary contraction during the isometric handgrip and sustain it for at least one minute.

### Cold pressor test (CPT)

The CPT was conducted after a 15-minute rest period after the IHT. Baseline SBP, DBP, MAP, HR, RR, and SpO_2_ measurements were recorded. Participants were then instructed to immerse the palm of their dominant hand into an ice-cold water bath, with water temperature ranging from 3 to 5°C, for one to two minutes. The immersion level was above the wrist [15]. If participants experienced discomfort or could not tolerate the cold, they were allowed to remove their hands from the water. During the CPT, SBP, DBP, MAP, HR, RR, and SpO_2_ parameters were simultaneously measured from the non-dominant hand.

### Statistical analysis

Data were meticulously checked and cleaned for accuracy before being entered into statistical software. Excel and SPSS 25.0 (V.25; IBM, Chicago, Illinois, USA) were used for data analysis. The study variables were analyzed using descriptive statistics, such as means, standard deviation, variations, frequency, and percentage distribution. Normality was assessed for continuous variables before statistical tests. An analysis of variance (ANOVA) test was performed to compare different cardiorespiratory parameters. A *P* value of 0.05 or less was considered significant.

## RESULTS

Table 1 shows the mean age and BMI of the 65 participants, with 69.2% being men and 30.8% women.

**Table 1:** Mean age and BMI of participants

Variables	*n*	Minimum	Maximum	Mean	Standard Deviation (SD)
Age	65	19.00	24.00	21.12	1.02
BMI	65	15.10	47.80	25.35	6.53

Table 2 summarizes the changes in physiological parameters—RR, SpO_2_, HR, SBP, DBP, and MAP—across three conditions: rest, IHT, and CPT. RR increased by 10.1% during IHT and 13.1% during CPT compared to the resting condition. The variations in RR between the conditions were statistically significant (*P* = 0.007). SpO_2_ levels remained stable across conditions, with no considerable change across all three conditions (*P* = 0.810).

**Table 2:** RR, SpO_2_, HR, SBP, DBP, and MAP responses during rest, IHT, and CPT tests

Parameter	Rest		IHT		CPT		*P* value
	Range	Mean ± SD	Range	Mean ± SD	Range	Mean ± SD	
RR	12.0–32.0	19.9 ± 4.2	12.0–40.0	21.9 ± 4.6	14.0–48.0	22.5 ± 5.6	0.007*
SpO_2_	94.0–99.0	97.8 ± 1.3	94.0–99.0	97.8 ± 1.2	90.0–100.0	97.9 ± 1.6	0.810
HR	50.0–120.0	83.0 ± 17.1	52.0–117.0	82.9 ± 15.1	47.0–119.0	80.1 ± 15.3	0.492
SBP	89.0–161.0	117.5 ± 15.8	82.0–161.0	114.2 ± 13.7	87.0–153.0	114.0 ± 14.2	0.300
DBP	47.0–110.0	76.6 ± 11.4	42.0–106.0	74.3 ± 11.1	43.0–103.0	75.3 ± 11.3	0.501
MAP	61.7–117.7	90.2 ± 11.4	62.3–115.0	87.6 ± 10.7	58.7–109.7	88.2 ± 11.0	0.381

* Significant differences

HR showed a marginal decrease of 0.1% from rest to the IHT and 3.5% from rest to the CPT, though these changes were not statistically significant (*P* = 0.492). SBP decreased by 2.8% during IHT and 3.0% during CPT compared to resting conditions, while DBP decreased by 3.0% during IHT and 1.7% during CPT, with no significant change (*P* = 0.501). MAP decreased by 2.9% during IHT and 2.2% during CPT compared to rest, with no significant difference (*P* = 0.381). These results highlight that RR increased progressively across the three conditions while HR and BP responses remained within controlled ranges. The results indicate that RR is the most responsive physiological parameter, with a statistically significant increase during IHT and CPT, reflecting heightened respiratory demand in these conditions. In contrast, SpO_2_ remained stable across all conditions, suggesting that oxygen supply was maintained despite increased physiological stress. HR and blood pressure parameters (SBP, DBP, and MAP) showed modest fluctuations. These findings imply that while RR is sensitive to stressors like IHT and CPT, other cardiovascular parameters remain relatively stable, maintaining homeostasis under these conditions.

**Figure 1:**
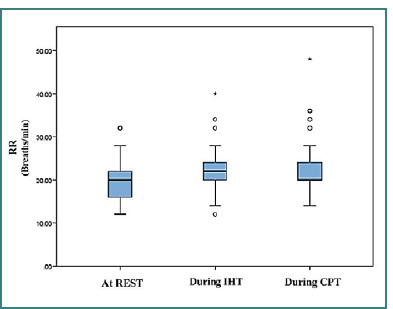
Respiratory rate at rest, during isometric handgrip test, and cold pressor test

Figure 1 displays the RR responses during rest, IHT, and CPT using box-and-whisker plots. The median RR at rest was lower than the values during IHT and CPT, indicating a progressive increase in RR across these conditions. The data show variability in all conditions, with a greater spread during IHT and CPT, including some outliers, particularly during CPT, where RR exceeded 40 breaths per minute. This suggests a more robust respiratory response during stress-inducing tasks like IHT and CPT than rest.

## DISCUSSION

This study investigated physiological parameters such as SBP, DBP, MAP, HR, RR, and SpO_2_ during rest, IHT, and CPT in healthy young Saudi Arabian adults. Only the RR showed a significant increase during both IHT and CPT. It has been reported that RR is a crucial vital sign that shows sensitivity to various pathological conditions [16]. Respiration is an automatic process governed by the autonomic nervous system [17]. The autonomic nervous system plays a crucial role in maintaining homeostasis and has been observed to function as a buffer against disruptions in hemodynamics [7]. A research report from India found that both normal and obese individuals experienced elevated HR, SBP, DBP, and MBP following IHT [6]. Similarly, a study from the United States reported increased values of these physiological parameters during CPT [15]. Both findings contradict the results of our research.

IHT can activate the cardiovascular system by stimulating efferent sympathetic pathways, increasing BP and HR [18]. However, this study did not observe a significant increase in SBP, DBP, MAP, and HR. The only parameter that showed a rise during IHT was RR. Researchers have identified that CPT activates afferent sensory pathways, initiating sympathetic responses that modulate the ANS [19]. A study from Nepal reported no significant rises in either SBP or DBP after the CPT, aligning with this study's outcomes. Similarly, they found a notable increase in RR in male and female subjects, which mirrors our observations. However, unlike our findings, their analysis revealed that only women exhibited a significant elevation in HR [20].

Studies reported that IHT and CPT activate the sympathetic nervous system [7,8]. The RR significantly increased among study participants during CPT and IHT, with no effects on other cardiovascular parameters or SpO_2_. IHT involves contracting the skeletal muscles in the dominant hand, which activates mechanoreceptors within these muscles. These mechanoreceptors are known to affect respiratory centers [21]. Clinical studies also support this perspective, showing that stimulation of limb skeletal muscles can aid respiratory rehabilitation in patients with spinal cord injuries [22].

According to researchers, CPT is recognized for activating pain fibers and eliciting autonomic responses [20]. Experimental studies have shown that the parabrachial nuclei, which receive input from somatic pain receptors, modulate cardio-respiratory responses to their activation [23]. Research has also demonstrated that passive observation of physical exercise can trigger muscle sympathetic nerve activity, suggesting a psychogenic origin [24].

We believe that the afferent inputs from the nociceptors during CPT and the activation of mechanoreceptors during IHT may have independently influenced respiratory center responses, resulting in an increased RR. Further research in this area could help uncover the precise mechanisms involved. The variations in physiological parameters observed during IHT and CPT in this study's subjects, compared to findings from different studies, could be attributed to several factors. Studies have shown that the modulation of pain and pain thresholds differs among ethnic groups [25]. Scientists have found that controlling BP and HR entails intricate processes affected by complex factors, such as ethnicity and gender [26]. Differences in autonomic regulation among various ethnicities become apparent early in life [27]. Research studies have reported variations in hand grip strength among different ethnic groups [28,29]. While we could not identify a precise physiological mechanism for the increase in RR during IHT, we speculate that the observed changes in RR may be attributed to ethnic differences in the Saudi Arabian subjects, which could have influenced the sensitivity of autonomic nerves, leading to an increase in RR. However, further research is required to investigate the underlying mechanisms.

Compared to other populations, the differences in physiological parameters in Saudi Arabian subjects undergoing IHT and CPT have not been explored. We propose that ethnicity may affect the sensitivity and vascular responses mediated by the autonomic nervous system during these tests. This hypothesis is supported by the variations in physiological parameters observed during IHT and CPT in studies conducted in various geographical regions [6,15].

The strength of this research is that it offers valuable insights into the complex interactions within the cardiorespiratory system during IHT and CPT. Tailoring experiments to specific physiological parameters is crucial for understanding these mechanisms. Different ethnic groups may exhibit varying susceptibility to cardiovascular and respiratory diseases. Recognizing ethnic-specific responses allows healthcare professionals to customize treatment strategies, improving outcomes for individuals from diverse backgrounds.

A limitation of this study is its restricted applicability to other populations due to the small sample size, consisting solely of young adult college students from Saudi Arabia. Additionally, psychological or emotional factors that may have influenced participants' test responses were not assessed. The study focused on cardiorespiratory parameters, such as RR, HR, SBP, DBP, MBP, and SpO_2_. However, incorporating other measures, like heart rate variability and more autonomic function markers, could offer deeper insights into physiological stress responses. We recommend structuring studies with various age groups and incorporating gender-specific analyses when comparing different ethnicities. By introducing new perspectives, this study can potentially deepen our understanding of the intricate mechanisms governing cardiorespiratory interactions.

## CONCLUSION

This research revealed that only the respiratory parameter, RR, significantly increased during IHT and CPT. It suggests the potential for ethnic disparities in autonomic regulation and highlights the need to customize cardiovascular health assessments and interventions for different populations. Specifically, the study sheds light on how healthy young Saudi Arabian subjects autonomically respond to physiological stressors, enhancing our understanding of cardiovascular health across diverse populations and guiding future interventions for improved cardiovascular outcomes worldwide.

## Data Availability

The data from this study are available from the corresponding author upon reasonable request.

## Personal thanks

The authors thank all the participants, administration, and support staff of KSAU-HS and KAIMRC Jeddah, Saudi Arabia, for their invaluable assistance in conducting this study.
